# Dobutamine, the chronotropic inodilator-venoconstrictor?

**DOI:** 10.1186/s40635-026-00944-y

**Published:** 2026-07-30

**Authors:** Rolf Erlebach

**Affiliations:** 1 Institute of Intensive Care Medicine, University Hospital Zurich, Zurich, Switzerland; 2 University of Zurich, Zurich, Switzerland

Dobutamine is classically understood through β1- and β2-adrenoceptor agonism [1], disregarding its effects on the venous circulation. In 1991, Binkley et al. [2] found that dobutamine still increased cardiac output in calves whose native ventricles had been replaced by pneumatically driven total artificial hearts — a model in which inotropic and chronotropic effects are eliminated by design. While marked systemic vasodilation was likely the main contributor, a concurrent rise in right atrial pressure (RAP) suggested additional reduced venous capacitance and augmentation of venous return. Because mean systemic filling pressure (MSFP) was not measured, vascular tone could not be separated into venous and arterial components.

The blood circulation is a closed series circuit. Therefore, flow can theoretically be interrogated at any point where measurement is feasible. However, this also means that cardiac output can be influenced at any part of the circulation, and isolating effects require consideration of venous, pulmonary vascular and systemic contributors. Characterization of the venous segment is challenging, because MSFP – the pressure in the systemic circuit in the absence of flow – cannot be measured directly while the heart is beating. Venous return, which equals cardiac output at steady state, is driven by the MSFP–RAP gradient. In Guyton’s framework, a rise in MSFP shifts the venous return curve rightward to a new equilibrium with higher cardiac output and higher RAP. RAP thus becomes a useful discriminator: When cardiac output increases, a concurrent rise in RAP implicates augmented venous return, whereas a fall suggests improved contractility or reduced afterload [3].

Applying this concept, Lai et al. [4] used stop-flow balloon occlusion of the right atrium to temporarily cease the circulation and measure MSFP across graded doses of dobutamine. The hemodynamic measurements in eight pigs were complemented by colloid infusion to estimate vascular compliance. Their methodology allowed them to provide a granular picture of the effects of dobutamine on the venous and systemic circulation. As expected, dobutamine raised cardiac output and lowered systemic vascular resistance. Notably, the observed increased pressure gradient for venous return resulted from elevated MSFP not reduced RAP. This finding complements the historical observations of Binkley et al. and provides further evidence that dobutamine decreases venous capacitance.

Targeting MSFP and venous return independently of inotropy and heart rate may be possible through modification of the racemic mixture of dobutamine and could have clinical relevance. Increasing stressed blood volume may be a desirable approach in septic shock, whereas in cardiogenic shock, further augmentation of preload is unlikely to provide benefit.

Important questions remain. Despite an increase in venous return and a decrease in systemic vascular resistance – two powerful forces that should raise stroke volume – it remained unchanged. To counterbalance these preload and afterload benefits, a concurrent reduction in contractility or tachycardia sufficient to limit filling time would be required. The marked tachycardia observed during dobutamine administration, together with the overall low RAPs, may partially explain these findings.

Taken together, this study demonstrates that understanding dobutamine’s hemodynamic effects requires moving beyond a purely cardiac-centric view toward a framework that incorporates the venous circulation with its determinants. Clinical translation could be accomplished using clinical settings that mimic the total artificial heart model, such as full veno-arterial extracorporeal membrane oxygenation support, where blood flow can be precisely controlled while direct cardiac effects of dobutamine are eliminated. Under these conditions, calculated estimates of mean systemic filling pressure appear reliable [5]. 

## Data Availability

Not applicable for this manuscript.
